# Data analysis issues for allele-specific expression using Illumina's GoldenGate assay

**DOI:** 10.1186/1471-2105-11-280

**Published:** 2010-05-26

**Authors:** Matthew E Ritchie, Matthew S Forrest, Antigone S Dimas, Caroline Daelemans, Emmanouil T Dermitzakis, Panagiotis Deloukas, Simon Tavaré

**Affiliations:** 1 Bioinformatics Division, The Walter and Eliza Hall Institute of Medical Research, 1G Royal Parade, Parkville, Victoria, 3052, Australia; 2 Wellcome Trust Sanger Institute, Wellcome Trust Genome Campus, Hinxton, Cambridge, CB10 1SA, UK; 3 Wellcome Trust Center for Human Genetics, University of Oxford, Roosevelt Drive, Oxford, OX3 7BN, UK; 4 Department of Genetic Medicine and Development, University of Geneva Medical School, 1 Rue Michel-Servet, Geneva, 1211, Switzerland; 5 Department of Obstetrics and Gynecology, Institute for Women's Health, University College London, 86-96 Chenies Mews, London, WC1E 6HX, UK; 6 Department of Oncology, University of Cambridge, CRUK Cambridge Research Institute, Li Ka Shing Centre, Robinson Way, Cambridge, CB2 0RE, UK

## Abstract

**Background:**

High-throughput measurement of allele-specific expression (ASE) is a relatively new and exciting application area for array-based technologies. In this paper, we explore several data sets which make use of Illumina's GoldenGate BeadArray technology to measure ASE. This platform exploits coding SNPs to obtain relative expression measurements for alleles at approximately 1500 positions in the genome.

**Results:**

We analyze data from a mixture experiment where genomic DNA samples from pairs of individuals of known genotypes are pooled to create allelic imbalances at varying levels for the majority of SNPs on the array. We observe that GoldenGate has less sensitivity at detecting subtle allelic imbalances (around 1.3 fold) compared to extreme imbalances, and note the benefit of applying local background correction to the data. Analysis of data from a dye-swap control experiment allowed us to quantify dye-bias, which can be reduced considerably by careful normalization. The need to filter the data before carrying out further downstream analysis to remove non-responding probes, which show either weak, or non-specific signal for each allele, was also demonstrated. Throughout this paper, we find that a linear model analysis of the data from each SNP is a flexible modelling strategy that allows for testing of allelic imbalances in each sample when replicate hybridizations are available.

**Conclusions:**

Our analysis shows that local background correction carried out by Illumina's software, together with quantile normalization of the red and green channels within each array, provides optimal performance in terms of false positive rates. In addition, we strongly encourage intensity-based filtering to remove SNPs which only measure non-specific signal. We anticipate that a similar analysis strategy will prove useful when quantifying ASE on Illumina's higher density Infinium BeadChips.

## Background

Preferential expression of one of the two alleles of a gene has been widely studied in the context of development, where key mechanisms such as genomic imprinting and X-inactivation lead to extreme allelic imbalances [1]. Allele-specific expression has been linked to the susceptibility of many human diseases [2-4].

Various experimental techniques exist for measuring ASE [5], including microarray-based approaches that have been used in a number of studies to screen for ASE in a high-throughput manner [6-11]. With microarrays, SNPs that fall within the coding regions of transcripts are used to quantify allelic imbalances in expression. Probes that distinguish between the signal from allele A and allele B in genomic DNA (gDNA) can be used to measure the relative amount of expression from each allele when mRNA (converted to cDNA) is hybridized to the array. Typically both gDNA and cDNA hybridizations are carried out on each sample. For individuals who are heterozygous (AB) at a particular SNP, which is usually determined by the gDNA hybridization, a distortion in the expected 1:1 ratio of allele A to allele B in the cDNA signal is an indication of ASE.

Illumina's two-color GoldenGate technology has been used to measure ASE in pancreatic cancer [10] and lymphoblastoid cell lines [12]. The GoldenGate assay applied to genotyping allows around 1500 SNPs to be investigated simultaneously in a Sentrix Array Matrix (SAM), which is made up of 96 separate arrays [13]. Each array contains around 30 replicate probes for each SNP. The assay consists of a PCR with universal primers that amplify DNA at the chosen loci to produce labelled material which is complementary to the appropriate 50 mer probe on the array at one end, and fluorescently labelled with either Cy5 (red) or Cy3 (green) dye depending on which nucleotide (allele A or allele B) is present. The relative signal for a given SNP provides a surrogate measure of the genotype, with high green intensity indicative of an AA genotype, high red intensity indicative of a BB genotype and an intermediate intensity in both channels an AB genotype. The GoldenGate assay allows for a custom panel of SNPs to be chosen for the array. As mentioned previously, these SNPs need to fall within a transcript to be useful for ASE profiling.

The fluorescence of each probe is quantified by Illumina's scanning software (BeadScan) and summarized values for each SNP are output by the BeadStudio software. The default preprocessing steps used in this analysis have been shown to offer good performance on spike-in data sets for Illumina's single-channel expression data [14]. In this paper, we investigate whether this holds true for two-color GoldenGate data. Along with the usual preprocessing steps of background correction [15], quality assessment and normalization [16], adjustment for dye effects [17,18] needs to be considered. Recent examinations of two-color data from Illumina's Infinium platform have revealed that normalization can reduce dye-bias [19].

In this paper, we focus on the data analysis issues that arise when Illumina GoldenGate BeadArrays are used to measure ASE. This paper is organized as follows. We first present the raw data from a series of arrays, and explore the general signal characteristics. Next, we examine a published control data set that allows us to quantify dye effects. We then look at the results from a mixture experiment, which is designed to produce known allelic imbalance at varying degrees for the majority of SNPs on each array, to assess the ability of different preprocessing methods to recover the true positives. Finally, we investigate what effect a gene's expression level has on our ability to measure ASE.

## Results and Discussion

### Signal characteristics and quality assessment

**Figure 1:**
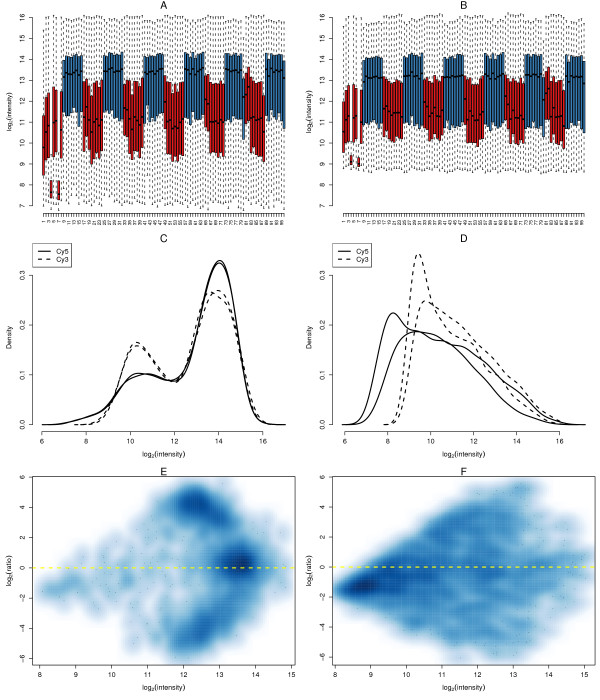
**Various plots of the raw signal from GoldenGate arrays measuring ASE**. Panels A and B show boxplots of the summarized log_2_(*Cy*5) and log_2_(*Cy*3) intensities respectively from a representative SAM. The data from each array were plotted in a separate boxplot, and color coded by sample (blue - gDNA, red - cDNA). Arrays 4 and 7 have low signal in both channels (IQR ≤ 1) and were excluded from downstream analysis. Density plots for each channel from two typical gDNA and cDNA arrays are presented in panels C and D respectively. These plots also show the systematic difference in overall signal between gDNA and cDNA hybridizations. Smoothed *MA*-plots for the gDNA (E) and cDNA (F) also highlight the differences. In these plots, a higher density of points is represented by a darker shade of blue.

Boxplots of the raw red and green intensities from a set of 96 arrays with both gDNA and cDNA hybridizations (Figure 1, panels A and B) show that the overall signal from the cDNA arrays is systematically lower than the signal from the gDNA arrays. Diagnostic plots such as this can be used to flag arrays with poor signal to exclude from further analysis; in Figure 1, we see that the 4th and 7th arrays have systematically lower signal over a compressed dynamic range compared to other arrays in the series. After examining these plots for many hundreds of arrays (data not shown), we find low interquartile range (IQR) of the log_2 _signal to be a good predictor of failed hybridizations, and use a threshold criterion of IQR ≤ 1 in either channel to flag poor quality arrays to exclude from further analysis [20,21].

Density plots of the intensities from each channel (Figure 1, panels C and D) show that the shape of the signal distribution depends on the sample type. For the gDNA arrays, the major signal peak occurs at higher intensities (Figure 1C), whereas for the cDNA arrays (Figure 1D), the reverse is true, with a peak at lower intensities. This has obvious implications for normalization; the cDNA and gDNA data must be treated separately given their very different signal characteristics.

The fundamental signal differences between the gDNA and cDNA hybridizations can also be seen by looking at their respective *MA*-plots (Figure 1, panel E and F) which display log-ratios (*M*-values) versus average intensities (*A*-values). For a typical gDNA array, three major clusters of points (one for each genotype: AA, AB and BB) can be seen in the *MA*-plot (Figure 1E). The data from a typical cDNA array (Figure 1F) is more diffuse, with a cluster of points occurring at low intensity, which presumably represents signal from SNPs in transcripts which are either non-expressed, or below the limits of detection using the GoldenGate technology.

### Dye effects

Although dye effects have been well characterized for spotted arrays, their existence for Illumina two-channel arrays has not been widely studied. By analyzing the summarized data from a dye-swap experiment, we assessed the magnitude of the dye effect for both gDNA and cDNA samples, and looked at whether within-array quantile normalization, as applied in other papers which analyze two-color data from various Illumina platforms [19,22], is beneficial.

**Figure 2:**
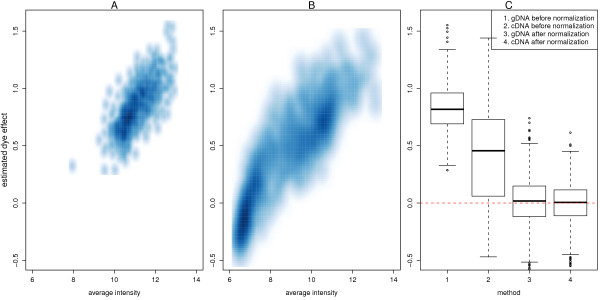
**Quantification of dye effects**. Smoothed scatter plots of the estimated dye effect from the SNP-wise linear models for the non-normalized gDNA (A) and cDNA (B) data versus average intensity are shown. In these plots, a higher density of points is represented by a darker shade of blue. For each analysis, the log-ratios are calculated as log_2_(*Cy*5/*Cy*3). Probes at lower intensities tend to have a bias towards the Cy3 channel (negative dye effect), while probes with higher intensities generally have a bias towards the Cy5 channel (positive dye-bias). Panel C shows the estimated dye effect before and after quantile normalization. The dye effect is systematically larger, and more variable before quantile normalization for both sample types. After quantile normalization, the dye effects are centered around zero.

SNP-wise linear models were fitted separately to the gDNA and cDNA log-ratios and average intensities. Each linear model summarizes the values from replicate arrays, and includes a global intercept (or *dye effect*) term, which measures the degree of asymmetry of the log-ratios when the dyes are swapped. Figure 2 (panels A and B) shows how the dye effect estimated for the non-normalized gDNA and cDNA data changes with average intensity. In these plots, there is a clear increasing trend for dye effect as average intensity increases. Probes at lower intensities tend to have a negative bias towards the Cy3 channel, while probes with higher intensities generally have a positive bias towards the Cy5 channel. Figure 2C shows the dye effects before and after within-array quantile normalization. After normalization, these effects are closer to zero and on a more comparable scale. This is desirable, since dye-bias represents a technical effect which is a nuisance variable for the purpose of measuring ASE.

For genotyping, the presence of dye-bias does not pose a problem, since the goal is to distinguish between three possible states (AA, AB or BB) which are generally well separated. For this application, the actual level of each group is mostly unimportant. However, when measuring ASE, dye-bias is of greater concern, as analysis methods typically search for systematic shifts in the heterozygous (AB) cDNA log-ratios away from the baseline heterozygote level inferred from the gDNA log-ratios. Such shifts are more likely to be driven by dye-bias in the absence of careful normalization. In addition, the magnitude of the dye effects need not be the same for RNA and DNA samples, and analysis methods which assume this may give rise to more false positives.

Although having dye-swap data allows us to model and correct for dye effects explicitly, in practice, this is not routinely possible using standard GoldenGate protocol. Hence throughout this paper, we have quantile normalized the data in an attempt to remove the dye effect as much as possible.

### Sensitivity and Specificity

**Figure 3:**
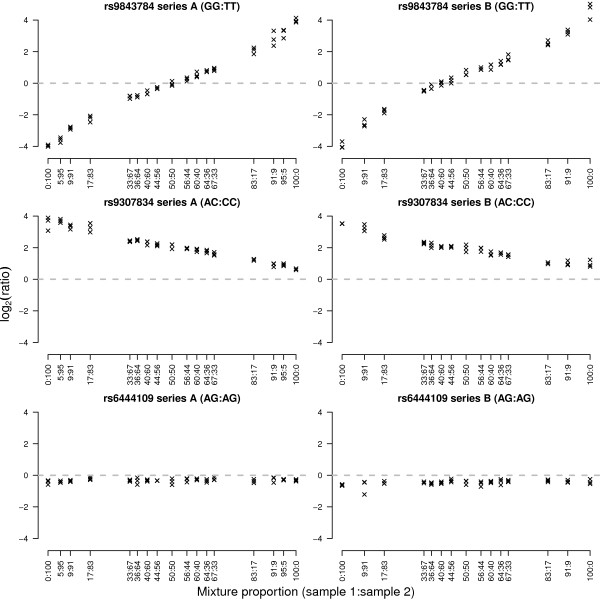
**Log-ratios for 3 SNPs from the mixture experiment**. The left and right hand columns show SNPs from series A and B respectively. Each series consists of similar titrations, which have each been replicated on 3 arrays. The data is ordered by increasing amount of sample 1 in the mixture (from 0:100 to 100:0). From top to bottom, we see SNPs which exhibit extreme ASE, intermediate ASE and no ASE respectively. Such SNPs provide the truth for our ROC analysis (Figures 4 and 5). The examples in the top and middle rows are true positives, of which there are 782 in series A and 808 in series B. The bottom example is a true negative, of which there are 533 in series A and 502 in series B.

The design of the mixture experiment produces known allelic imbalances. SNPs which are of the same genotype in the different pooled individuals form the true negative set, while SNPs with different genotypes are true positives for allelic imbalance. The mixture experiment we analyze is made up of two independent series (A and B) which pool DNA from different pairs of individuals (see Methods). Figure 3 shows examples of true positives (top and middle panel) and true negatives (bottom panel) for allelic imbalance. When both individuals are homozygous for different alleles at a given SNP (top panel), we see a trend from large positive or negative log-ratios at the extreme 100:0 and 0:100 mixtures, which get closer to zero as the mixtures become more even in concentration (50:50). This class of SNPs are the easiest to detect, as they exhibit allelic imbalance over a large range. The second class of true positives are SNPs which are homozygous in one individual and heterozygous in the second (Figure 3, middle panel). These SNPs are more difficult to measure changes for than the first category, since their allelic imbalance occurs over a compressed dynamic range. Finally, the true negative cases are SNPs for which both individuals have the same genotype (Figure 3, bottom panel). Alterations in the mixing proportion does not alter the ratio of allele A to allele B for these SNPs.

**Figure 4:**
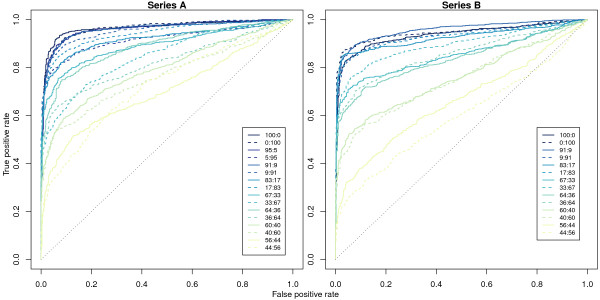
**ROC plots from the mixture experiment**. Data from series A (left) and series B (right) are shown for each mixture. A clear and not unexpected trend seen here is for the true positive rate to decrease as the mixture proportions become closer together. The 56:44 and 44:56 mixtures show the lowest true positive rates across the range of false positive rates, while the most extreme mixtures (0:100, 100:0, 5:95, 95:5) produce the highest true positive rates. The true positive set of SNPs (782 for series A and 808 for series B) were those with different genotypes in the two individuals whose DNA was mixed. The true negative set of SNPs (533 for series A and 502 for series B) consisted of those with the same genotype in the two individuals.

The built-in truth for each SNP from our mixture experiment, along with access to the raw data, allows us to measure the sensitivity and specificity of different preprocessing options applied to the data. In Figure 4, we see the Receiver Operator Characteristic (ROC) curves for series A and B for each of the mixtures calculated using the true positives and true negatives determined *a priori *using the independent HapMap genotypes for each pair of individuals (see Figure 3 and Methods). Each curve plots the sensitivity versus specificity of recovering SNPs with known allelic imbalance as the log-odds of detection is varied.

What is clear, and not unexpected from this analysis, is that the true positive rate declines as the mixing proportions of the samples become more even. This implies that GoldenGate can detect larger allelic imbalances more confidently than more subtle changes, which are more difficult to distinguish from experimental noise. For all mixtures down to the most similar 56:44 and 44:56 comparisons (which corresponds to subtle absolute fold-changes of around 1.3), our analysis (see Methods) produces better results than selecting SNPs at random.

**Figure 5:**
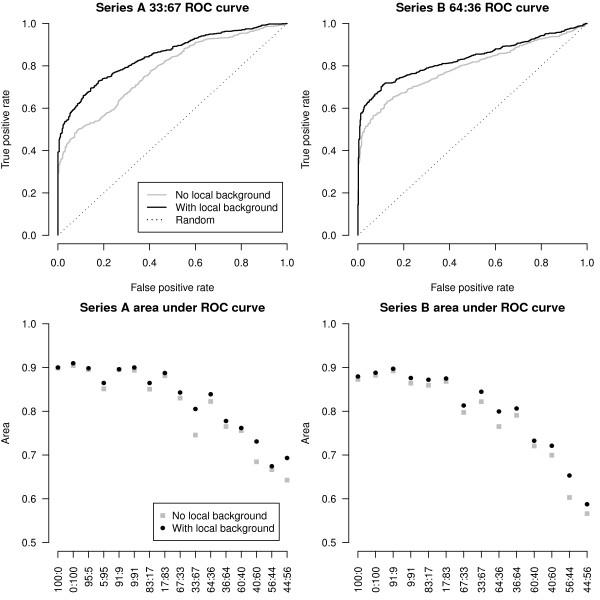
**A comparison of different background correction methods**. ROC plots for data processed with and without local background correction for the series A 33:67 mixture (top left) and series B 64:36 mixture (top right) are shown. These curves were calculated using the relevant true positive and true negative sets outlined in Figure 3 and Methods. In both cases, locally background corrected data (black line) offers more true positives than data which has not been background corrected (gray line). Looking at the results from all mixtures, we see that for series A (bottom left) and series B (bottom right), local background correction gives more true positives in almost all situations when performance is measured using area under the ROC curve.

We next look at the effect of Illumina's local background subtraction on the true positive rate. Figure 5 shows ROC curves for the 33:67 mixture from series A (top left) and the 64:36 mixture from series B (top right) which show that local background correction offers systematically better performance, delivering more true positives compared to not background correcting the data. For nearly all mixtures, the area under each ROC curve is larger when local background subtraction has been applied (Figure 5, bottom left and bottom right), representing a global performance gain.

### Intensity-based filtering

The method used to detect ASE in Tan *et al*. (2008) [10] and Serre *et al*. (2008) [12] involves linear interpolation of the AB heterozygote signal from the AA and BB homozygote log-ratios. Briefly, for each SNP, the center (median or mean), upper (median + 2 MADs or mean + 2 SDs) and lower (median - 2 MADs or mean - 2 SDs) confidence intervals are calculated using the cDNA log-ratios from the AA and BB genotypes respectively. This calculation is repeated for the gDNA log-ratios. Next, the cDNA upper confidence intervals are regressed against the gDNA lower confidence intervals and the cDNA lower confidence intervals are regressed against the gDNA upper confidence intervals. The respective centers for the homozygous genotypes are also regressed against each other. These regression lines provide upper and lower limits. ASE is called when the observed cDNA log-ratio from a heterozygous individual falls above or below the interpolated upper or lower value obtained using the gDNA log-ratio from the same individual.

**Figure 6:**
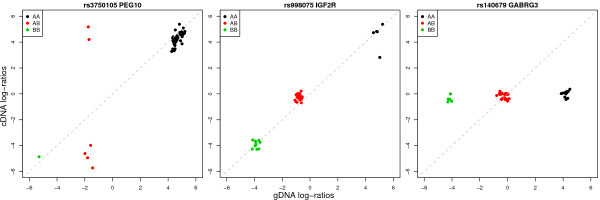
**Plots of cDNA versus gDNA log-ratios for 3 SNPs located in known imprinted genes**. Averaged log-ratios from the 44 CEU samples in Tan *et al*. (2008) are shown. The first example (PEG10) provides a clear example of ASE, with the AB cDNA log-ratios (red) at similar levels to either the AA, of BB cDNA log-ratios, which is indicative of silencing. For this SNP, there is a clear linear trend between the homozygous cDNA and gDNA log-ratios. In the second example (IGF2R), there is again a linear trend, although in this case, there is no evidence for ASE, with the AB log-ratios around zero. In the final example (GABRG3), there is no obvious linear trend. This SNP provides an example of non-specific signal, where the cDNA log-ratios lie around zero irrespective of the alleles present.

After examining many plots of cDNA log-ratios versus gDNA log-ratios, it is clear that for some probes there is a strong linear relationship between these values (Figure 6, panels A and B). In these situations, ASE can be detected. There are also many examples where the two alleles cannot be clearly differentiated in the cDNA samples, as shown in Figure 6C. In this plot, the presence of allele A or allele B does not produce a noticeable difference in the homozygous cDNA log-ratios.

**Figure 7:**
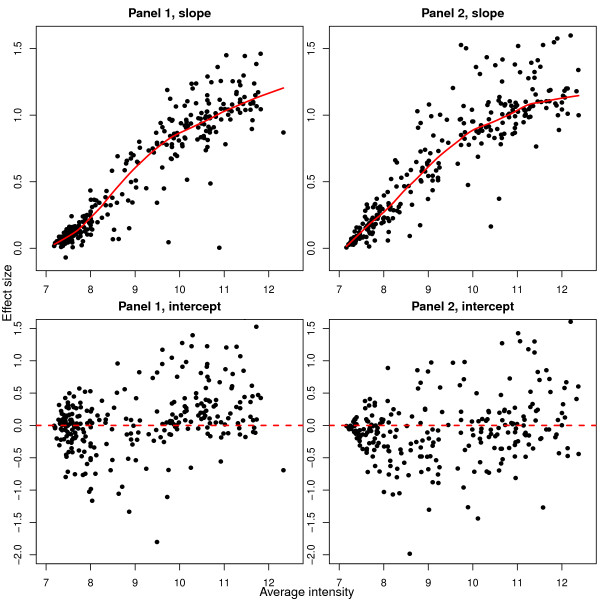
**Intensity trends in slope and intercept for the regression analysis between the cDNA and gDNA log-ratios**. The slopes and intercepts were calculated from the data in Tan *et al*. (2008) for SNPs with at least 3 AA and 3 BB individuals from the complete set of 142 samples. The average intensity for each SNP (calculated using the same data) is plotted on the x-axis. In total 277 SNPs are plotted from panel 1 and 261 from panel 2. Log-ratios were calculated after within-array quantile normalization of the Cy5 and Cy3 intensities. This figure shows an increasing trend for slope as average intensity increases. There is no such trend for intercept.

To explore this phenomenon, we fitted a separate linear model for each SNP, which regressed the average cDNA log-ratio from each individual against the average gDNA log-ratio for the homozygotes (see Methods). This analysis summarizes the information displayed in Figure 6 into two values per SNP, a slope and an intercept. Figure 7 shows the slope or intercept versus average intensity calculated across all samples for the different SNP panels in Tan *et al*. (2008). For slope of the regression line, we see a clear increasing trend as average intensity increases. For intercept, there is no strong intensity-based trend.

**Figure 8:**
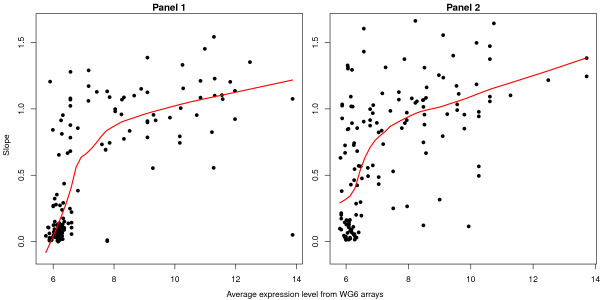
**Intensity trends in slope for the regression between the cDNA and gDNA log-ratios where average intensity has been ascertained using a different microarray platform**. Slopes from the regression of cDNA log-ratios against gDNA log-ratios from the 44 CEU individuals in Tan *et al*. (2008) for SNPs with at least 3 individuals of each homozygous genotype (AA, BB) versus average intensity measured on the Illumina WG-6 arrays from Stranger *et al*. (2007) are shown. In panel 1, 155 SNPs are plotted and in panel 2, 152 SNPs are shown. As observed in Figure 7, we see a trend for increasing slope with increasing intensity.

A similar relationship also holds when average intensity is quantified using a different microarray platform (Figure 8). For each transcript interrogated for ASE in the CEU (Centre d'Étude du Polymorphisme Humain samples collected from UT, USA) samples in Tan *et al*. (2008), an average expression level across the CEU series from Stranger *et al*. (2007) [23] was calculated. This data set measured expression in the same lymphoblastoid cell lines from CEU individuals using a different platform (Illumina WG-6 microarrays).

Intuition would suggest that the strength of the regression line should be related to the overall expression level of the transcript in which the coding SNP falls. Lowly expressed, or non-expressed transcripts provide little or no starting template for the GoldenGate assay to PCR amplify and label, which produces either weak signal or signal that is pure noise. This results in a low slope in our regression analysis. Figure 8 indicates that this is not due to the GoldenGate protocol working less well for these transcripts, as a similar trend can be seen when expression level is quantified using an independent array platform.

In light of these observations, we have found it useful to remove SNPs with average intensity below a particular threshold [20,21]. This has the effect of removing SNPs with non-specific allele A and allele B signals, which should reduce the number of false positives obtained by an appropriate ASE testing procedure. Figure 7 can be used to select this threshold; for lower cut-offs, more SNPs with non-specific signal (low slope) will be analyzed. In general the higher the average intensity, the greater the ability to distinguish between the two alleles. The cut-off can be adjusted depending upon the stringency desired.

## Conclusions

Our survey of ASE experiments which use the Illumina GoldenGate platform has highlighted a number of important data analysis issues to consider. Analysis of a dye-swap data set generated in-house by Illumina reveals significant dye effects in the log-ratios of both gDNA and cDNA hybridizations prior to normalization. Applying within-array quantile normalization reduces this effect considerably, and is recommended in analyses of data from the GoldenGate platform.

Our mixture data set showed that ASE can be detected more reliably when the imbalances are large, with the true positive rate diminishing fairly monotonically as the mixtures get closer together (down to 56:44 or approximately 1.3 fold). This experiment provides an overestimate of how well the GoldenGate assay will perform in practice, as the pooling of gDNA samples ensures a relatively constant amount of template is available for each SNP as input to the assay. In cDNA samples, this amount will vary depending on the expression level of the transcript. In Serre *et al*. (2008), imbalances down to 60:40 or 1.5-fold could be distinguished from experimental noise in cDNA samples. To measure smaller changes, other technologies such as second-generation sequencing methods [24,25] are likely to be more sensitive.

We find that the default background adjustment performed by Illumina improves the detection of true ASE using our control data. The benefit of local background subtraction has also been shown in analyses of control data from Illumina's single-channel expression arrays [14]. The need to apply intensity-based filtering to remove non-responding SNPs was also highlighted. Throughout this paper, we have used linear models and the *limma *package to summarize data from replicate hybridizations and derive test statistics for ASE. When replicate data are not available, other tests may be more appropriate, such as SNP-wise tests for increased variation in heterozygote log-ratios in cDNA versus gDNA samples (Mark Dunning, personal communication). While the dye-bias issue is Illumina-specific, the remaining points raised in this paper are likely to be pertinent when other array-based technologies are used to measure ASE. A major limitation of GoldenGate is that it only allows a relatively small number of genes to be surveyed for ASE per panel. Current higher density microarrays, which genotype around 1 million SNPs per array, will allow studies to scale up genome-wide. The majority of SNPs on these arrays, which fall in non-coding regions, will however be non-informative for ASE.

A final consideration when analyzing ASE using microarrays is the impact copy number variation will have on the signal. In general, genotype calling methods assume three distinct clusters for each SNP (AA, AB, BB) in the gDNA signal. In the presence of copy number variation, there may be additional clusters which will cause problems for standard genotype calling methods. Incorrect genotypes can lead to misleading results, as the calls play an important role in any test for ASE, which can only be ascertained at heterozygous loci. Bearing this in mind, we recommend that ASE calls in copy number variable regions be carefully scrutinized to avoid false positives.

## Methods

### Data sets

Four ASE data sets were analyzed in this paper. First, one SAM that included 48 gDNA and 48 cDNA arrays from the CEU samples in Dimas *et al*. (2008) [20] were analyzed to obtain a preliminary view of the data (Figure 1). The raw data from this experiment are available in the ArrayExpress database [26] under accession number E-TABM-927.

The second data set was the dye-swap experiment from Tan *et al*. (2008) [10], which consisted of cDNA and gDNA samples from 3 HapMap individuals hybridized in duplicate using both regular and dye-swapped chemistry. Data from this set of 24 arrays were provided by Aik Choon Tan (personal communication).

The third data set was from a mixture experiment. The raw data from this experiment are available in the ArrayExpress database [26] under accession number E-TABM-855. Arrays containing the same custom SNP panel as Dimas *et al*. (2008) [20] were used. Two series (A and B) were generated using different pairs of HapMap individuals. For each pair, individuals were selected from the CEU and YRI populations that had the greatest differences for as many SNPs as possible from the custom panel. In series A, gDNA from HapMap individuals NA12892 and NA19092 were mixed in the following proportions: 0%:100%, 5%:95%, 91%:9%, 83%:17%, 67%:33%, 64%:36%, 60%:40%, 56%:44%, 50%:50%, 44%:56%, 40%:60%, 36%:64%, 33%:67%, 17%:83%, 9%:91%, 5%:95% and 100%:0%. In series B, gDNA from individuals NA07022 and NA19143 were pooled in the following proportions: 0%:100%, 91%:9%, 83%:17%, 67%:33%, 64%:36%, 60%:40%, 56%:44%, 50%:50%, 44%:56%, 40%:60%, 36%:64%, 33%:67%, 17%:83%, 9%:91% and 100%:0%. Genotypes for each SNP were downloaded from HapMart [27]. For SNPs that were either homozygous and different (AA:BB or BB:AA), or heterozygous and homozygous (AA:AB, BB:AB, AB:AA or AB:BB) in a given pair of individuals, allelic imbalances should be present. These SNPs (782 in series A and 808 in series B) form our true positive set. SNPs which have the same genotype for each individual (AA:AA, BB:BB or AB:AB) should not change with mixing concentration. These SNPs (533 in series A and 502 in series B) make up the true negative set. SNPs with missing data in HapMart (NN) were excluded from the analysis (15 in series A and 20 in series B), as were SNPs with IDs which could not be found in HapMart (206). Each mixture was hybridized in triplicate using the experimental protocol described in Dimas *et al*. (2008) [20].

The HapMap and Pancreatic cancer data from Tan *et al*. (2008) [10] were also analyzed. Duplicates from 142 individuals and 2 panels of markers (which we name panel 1 and 2 and contain 927 and 1188 SNPs respectively) were analyzed.

To measure expression of each gene independently, the Illumina WG-6 expression data from Stranger *et al*. (2007) [23] were downloaded. These arrays use both different probes and chemistry to measure the level of gene expression compared to GoldenGate in many of the same samples (14 out of 42). The normalized CEU intensities were averaged across all samples to obtain an average expression level for each probe. Probes were matched between platforms using gene symbols.

### Data preprocessing

The bead-level data from each array in the mixture experiment were summarized by calculating the per bead type average of 4 quantities after outlier removal both with and without the local background estimates subtracted: the log_2_(*Cy*3) and log_2_(*Cy*5) intensities, average log-intensities () and log-ratios (*M *= log_2_(*Cy*5/*Cy*3)). For each quantity, outlier beads (those with values more than 3 MADs above or below the median) were removed prior to calculating the average. The local background intensities were estimated using an average of the five dimmest pixels within the 17 × 17 pixel area around each bead centre, as per Illumina's default image analysis.

To obtain normalized data, the summary log_2_(*Cy*3) and log_2_(*Cy*5) values from each array were quantile normalized (within-array) and log-ratios were calculated. This analysis was carried out in R [28] using the *beadarray *[29] and *beadarraySNP *packages.

BeadStudio output from the dye-swap experiment in Tan *et al*. (2008) was provided by Aik Choon Tan (personal communication). For each array, log-ratios and average log-intensities were calculated both with and without quantile normalization between channels.

The remaining data from Tan *et al*. (2008) were downloaded from the GEO database [30] using the *GEOquery *Bioconductor package [31]. This data set consisted of replicate arrays of both gDNA and cDNA samples for 142 individuals. The red and green intensities from each array were quantile normalized, and log-ratios and average log-intensities were calculated for each SNP on each array.

The two-dimensional smoothed scatter plots (Figure 1, panels E and F and Figure 2, panels A and B) were generated in R [28] using the *smoothScatter *function with the default options.

### Linear model analysis

To summarize the data from replicate arrays, SNP-wise linear models were fitted to the log-ratios [32] using the *limma *package [33]. For each experiment, the models were fitted separately for the cDNA and gDNA data sets. For SNP *i*, we can write the linear model as(1)

where **y**_*i *_= (*y*_*i*1_, ..., *y*_*iN*_)^*T *^is the vector of log-ratios from arrays 1, ..., *N*, *X *is a known design matrix with full column rank, and ***β***_*i *_= (*β*_*i*1_, ..., *β*_*iK*_)^*T *^is a SNP-specific vector of regression coefficients.

The linear model assumes(2)

where  is an unknown factor. We assume the log-ratios *y*_*ij *_are normally distributed and that the values from different arrays are independent. Ordinary least squares estimators of ***β***_*i *_() and  (, residual mean square) were obtained for each SNP.

For the mixture experiment, contrasts given by ***α***_*i *_= **C**^*T*^***β***_*i*_, where **C **is a contrast matrix which gives all pair-wise comparisons between a given mixture and the 50:50 mixture. This corrects for systematic dye-biases or genotype effects, which shift the baseline away from 0.

Moderated *t*-statistics were calculated using the empirical Bayes shrinkage procedure of Smyth (2004) [32] to test the null hypothesis *α*_*ik *_= 0. Since the mixture experiment uses samples from individuals with known genotypes, we know *a priori *which SNPs will have a differential allelic response. Sensitivity and specificity were calculated for the concentrations in each series by ranking SNPs by their log-odds.

For the dye-swap experiment, SNP-wise regression models as described above (Equation 1) which included an intercept (dye effect) term were fitted separately for the log_2 _cDNA and gDNA log-ratios, both before and after within-array quantile normalization.

### Regression between cDNA and gDNA log-ratios

We assessed the degree of linear trend between the cDNA and gDNA log-ratios from Tan *et al*. (2008) [10] (Figure 6) more globally by regressing the average cDNA log-ratios from the above linear models (Equation 1) against the average gDNA log-ratios for the homozygous individuals only. The model included both a slope and intercept term that was separately estimated for each SNP. For Figure 7, these regression parameters are plotted versus average intensity (estimated using the same arrays) for SNPs with at least 3 AA and 3 BB homozygotes (277 in panel 1 and 261 in panel 2). The requirement for at least 6 observations ensured that the slope and intercept terms were reasonably well estimated. This analysis was repeated for the CEU individuals only in Figure 8, which shows the slope for 155 SNPs from panel 1 and 152 from panel 2 versus the average intensity calculated from WG-6 expression arrays.

## Abbreviations

The following is a summary of the abbreviations used in this paper: **ASE**: allele-specific expression; **CEU**: Centre d'Étude du Polymorphisme Humain samples collected from UT, USA, which are part of the HapMap project [34,35]; **MAD**: median absolute deviation; **PCR**: Polymerase chain reaction; **ROC**: Receiver Operator Characteristic, a method used to assess sensitivity and specificity; **SAM**: Sentrix Array Matrix, a collection of 96 BeadArrays in 96-well plate format; **SNP**: Single Nucleotide Polymorphism; **WG-6**: whole-genome, expression BeadChips from Illumina which contain 6 individual BeadArrays; **YRI**: samples from individuals from Yoruba in Ibadan, Nigeria, which are part of the HapMap project [34,35].

## Authors' contributions

MER performed the analysis and drafted the manuscript, MSF performed the experiments, MSF, AD, CD, ETD and PD planned the experiments and ST supervised the research and finalized the manuscript. All authors read and approved the final manuscript.
